# Maimendong decoction and its active ingredient, ophiopogonin D, alleviate bleomycin-induced pulmonary fibrosis by regulating the behavior of lung fibroblasts

**DOI:** 10.1186/s13020-025-01206-x

**Published:** 2025-08-29

**Authors:** Mingjie Yang, Xinyue Zhang, Shengchuan Bao, Shusen Yang, Yilin Zhang, Yushan Liu, Chengjun Li, Junbo Zou, Jingtao Li, Shuguang Yan

**Affiliations:** 1 College of Basic Medicine, Shaanxi University of Chinese Medicine, Xianyang, 712046 China; 2 Key Laboratory of Gastrointestinal Diseases and Prescriptions in Shaanxi Province, Shaanxi University of Chinese Medicine, Xianyang, 712046 China; 3 Department of Internal Medicine, The Affiliated Hospital of Wuhan Sports University, Wuhan, 430000 China; 4 Department of First Clinical Medical College, Shandong University of Traditional Chinese Medicine, Jinan, 250000 China; 5 Department of Gastroenterology, the First Affiliated Hospital of Xi’an Jiaotong University, Xi’an, 710061 China; 6 College of Pharmacy, Shaanxi University of Chinese Medicine, Xianyang, 712046 China; 7 Departments of Infectious Disease, The Affliated Hospital of Shaanxi University of Chinese Medicine, Xianyang, 712000 China

**Keywords:** Idiopathic pulmonary fibrosis, Maimendong decoction, Ophiopogonin D, Bleomycin, Ferroptosis

## Abstract

**Background:**

Idiopathic pulmonary fibrosis (IPF) is a chronic lung disease characterized by progressive scarring of lung tissue, which can lead to respiratory failure and other serious complications, making the search for safe and effective therapeutic agents an urgent priority; Maimendong Decoction (MMDD), a traditional Chinese herbal formula widely employed in clinical practice to treat pulmonary disorders, has been shown to reduce inflammatory cytokines, suppress pro-fibrotic factors, and alleviate oxidative stress, yet the precise mechanisms by which MMDD exerts its anti-fibrotic effects remain unclear.

**Purpose:**

This study aims to evaluate the therapeutic efficacy of MMDD in pulmonary fibrosis and to elucidate its underlying mechanisms.

**Methods:**

Using a bleomycin (BLM)-induced mouse model of idiopathic pulmonary fibrosis, we assessed the therapeutic impact of MMDD on pulmonary fibrosis; concurrently, Ophiopogonin-D (OP-D), a principal active ingredient of MMDD, was evaluated in an in vitro human fetal lung fibroblast model treated with transforming growth factor-β (TGF-β) to elucidate its precise anti-fibrotic mechanism; finally, multi-omics technologies and bioinformatics analyses were employed for comprehensive validation.

**Results:**

MMDD ameliorated the degeneration observed in BLM-induced pulmonary fibrosis by curbing fibroblast activation and the over-deposition of extracellular matrix. Comprehensive multi-omics and bioinformatics analyses further indicated that MMDD modulates fibroblast behaviors—including proliferation and ferroptosis—through inhibition of the TGF-β signaling pathway. In vitro studies showed that OP-D induces ferroptosis in TGF-β-treated lung fibroblasts by blocking the negative regulatory signals of ferroptosis, mainly via downregulation of Ferritin Heavy Chain 1 (FTH1) expression. Moreover, overexpressing FTH1 nullified the anti-fibrotic activity of OP-D.

**Conclusion:**

The results of this study indicate that MMDD and its active component, OP-D, can treat IPF by targeting the proliferation of lung fibroblasts.

**Supplementary Information:**

The online version contains supplementary material available at 10.1186/s13020-025-01206-x.

## Introduction

Idiopathic pulmonary fibrosis (IPF) is a chronic, progressive interstitial lung disease characterized by relentless fibrosis that leads to pulmonary scarring, respiratory failure, high mortality, and poor prognosis [1]. Current therapies such as nintedanib and pirfenidone can slow disease progression but cannot reverse established fibrosis and are often associated with adverse effects including anorexia, gastrointestinal disturbances, and hepatotoxicity, which reduce patient compliance [2]. Although the precise cause of IPF remains unclear, abnormal proliferation and differentiation of lung fibroblasts together with excessive extracellular matrix (ECM) deposition are central to its pathogenesis. In this process, fibroblasts proliferate unchecked, differentiate into contractile myofibroblasts that secrete fibrotic mediators and ECM, and upregulate α-smooth muscle actin (α-SMA) expression, driving ECM accumulation [3, 4]. Therefore, inhibiting fibroblast proliferation and differentiation is a promising therapeutic strategy for pulmonary fibrosis, yet no drugs targeting this pathway are currently available.

Mai Men Dong Decoction (MMDD) is a classical TCM formula first recorded in the Jingui Yaolue and consists of six herbs: *Ophiopogon japonicus* (Linn. f.) Ker Gawl., *Pinellia ternata* (Thunb.) Breit., *Glycyrrhiza uralensis* Fisch., *Panax ginseng* C.A.Meyer, *Oryza sativa* Linn. and *Ziziphus jujuba* Mill. For over two thousand years, MMDD has been used to nourish lung and stomach yin, direct rebellious qi downward, and treat “Fei Wei,” a syndrome of chronic cough, sputum, dyspnea, and internal heat from yin deficiency that corresponds to modern chronic pulmonary disorders [5]. Clinically, MMDD has shown efficacy in pulmonary fibrosis by inhibiting fibroblast activation, reducing oxidative stress, and exerting anti-inflammatory effects [6]. Its major active components have well-characterized actions: Ophiopogonin-D (OP-D) inhibits lung fibroblast activation via the TGF-β1/Smad and PI3K/AKT pathways [7]; Ginsenoside Rg3 attenuates pulmonary fibrosis by blocking endothelial-to-mesenchymal transition and mitigates liver fibrosis by epigenetically upregulating ACSL4 to promote ferroptosis in stellate cells [8, 9]; Pinellia ternata reduces airway inflammation and mucus secretion [10, 11]; Glycyrrhiza uralensis extracts prevent and treat pulmonary fibrosis and inflammation in rats [12]; Ziziphus jujuba extracts alleviate bleomycin-induced fibrosis via the TGF-β1/Smad pathway [13]; and resistant starch from Oryza sativa mitigates renal fibrosis by inhibiting inflammation and TGF-β activation [14]. Together, these data suggest MMDD may treat IPF by suppressing lung fibroblast proliferation and differentiation, although the precise molecular mechanisms remain to be fully elucidated.

To evaluate our hypothesis, we used a mouse model of IPF induced by bleomycin to assess the pharmacodynamic effects of Maimendong Decoction (MMDD). We combined transcriptomic and proteomic analyses with high performance liquid chromatography and UPLC-MS/MS to identify MMDD’s core active compounds and their key molecular targets. We then validated these compounds and targets through transcriptomic profiling and molecular biology experiments to elucidate the essential components and mechanisms by which MMDD exerts therapeutic effects in IPF.

## Materials and methods

### Reagents and materials

MMDD consists of six medicinal herbs, all supplied by Shaanxi Xingshengde Pharmaceutical Co., Ltd. (Xi’an, China) (Table 1). Bleomycin and prednisone were obtained from Selleck Chemicals (Houston, USA) and Xianju Pharmaceutical (Taizhou, China), respectively. Ophiopogonin-D (OP-D) was purchased from Chenguang Biology Co., Ltd. (Baoji, China). Assay kits for hydroxyproline, glutathione (GSH), reactive oxygen species (ROS), malondialdehyde (MDA), and apoptosis detection were sourced from Jiancheng Bioengineering (Nanjing, China), Keygen Biotech (Nanjing, China), and Dojindo Biotech (Shanghai, China). Human fetal lung fibroblasts were acquired from the BeNa Culture Collection (Beijing, China). Deferoxamine mesylate (DFO; Cat. No. S5742) was also purchased from Selleck Chemicals (Houston, USA).

**Table 1:** Composition of MMDD decoction

Latin name	English name	Local name	Used part	Weight (g)
Ophiopogonis Radix	Dwarf Lilyturf Root Tuber	Maidong	Root tuber	42 g
Pinelliae Rhizoma	Pinellia Tuber	Banxia	Rhizome	6 g
Oryzae Semen	Non-glutinous Rice	Jingmi	Seed	6 g
Ginseng Radix et Rhizoma	Ginseng Root and Rhizome	Renshen	Root and rhizome	9 g
Glycyrrhizae Radix	Licorice Root	Gancao	Root	6 g
Jujubae Fructus	Jujube Fruit	Dazao	Fruit	24 g

### Ultra-performance liquid chromatography tandem mass spectrometry (UPLC–MS/MS) analysis

MMDD was analyzed by UPLC-MS/MS on an Agilent SB-C18 column (1.8 µm, 2.1 × 100 mm) using 0.1% formic acid in water (A) and acetonitrile (B) as the mobile phase. The gradient elution began at 95% A/5% B, ramped to 5% A/95% B over 9 min (held for 1 min), then returned to 95% A/5% B in 1.1 min (held for 2.9 min). The flow rate was 0.35 mL/min, column temperature 40 °C, and injection volume 2 µL. Data were acquired on an ESI-QTRAP mass spectrometer.

### In vivo experiments

#### Animal grouping and modeling

The study utilized SPF-grade male C57BL/6 mice, weighing 20 ± 2 g, sourced from GemPharmatech (Chengdu) Co., Ltd. (Chengdu, China). Ethical approval was obtained from the Ethics Committee of Shaanxi University of Chinese Medicine (Approval Number: SUCMDL20211010023), and all experimental procedures were conducted in accordance with the NIH Guide for the Care and Use of Laboratory Animals (NIH Publication No. 85-23, revised 1985). The mice were randomly divided into six groups (*n* = 10 per group): control, bleomycin (BLM)-induced model, high-dose Maimendong Decoction (MMDD-H), medium-dose MMDD (MMDD-M), low-dose MMDD (MMDD-L), and positive control (prednisone). Except for the control group, all mice received a single intratracheal injection of BLM (5 mg/kg). Starting from the third day after BLM administration, the mice were gavaged daily with MMDD at high (17.16 mg/kg), medium (8.58 mg/kg), or low (4.29 mg/kg) doses, or with prednisone (5 mg/kg) at a volume of 0.2 mL for a period of 28 days. At the end of the study, serum and lung tissues were collected for further analysis.

#### Detection of hydroxyproline

Approximately 0.2 g of lung tissue was weighed, finely minced, and mixed with 2 mL of extraction solvent. The mixture was boiled, cooled, adjusted to pH 6.0–8.0, diluted with distilled water, and centrifuged at 16,000 rpm for 20 min; the resulting supernatant was then used to determine the hydroxyproline concentration.

#### Hematoxylin and eosin (H&E) staining

Lung tissues were dehydrated through a graded ethanol series, cleared with a clearing agent, embedded, sectioned, slide-baked, and dewaxed. Sections were stained with Mayer’s hematoxylin for 5 min, then rinsed under tap water until they turned blue. They were subsequently stained with 1% water-soluble eosin for 5 min, followed by a tap water rinse. Finally, the slides were mounted with neutral gum and examined and photographed under a microscope.

#### Masson staining

After dewaxing, sections were stained with Weigert’s iron hematoxylin for 5 min, differentiated in 1% hydrochloric acid in ethanol for 3 s, and counterstained with acid fuchsin-Ponceau for 3 min, with each step followed by a brief rinse under running water. The sections were then differentiated in 1% phosphomolybdic acid and stained with aniline blue for 5 min each, rinsed in 1% glacial acetic acid for 1 min, dehydrated, and mounted with neutral gum. Finally, the slides were examined and photographed under a microscope.

#### Sirius red staining

After deparaffinization, sections were stained with Harris’ hematoxylin for 5 min and rinsed with distilled water. They were then stained with picric acid-Sirius red for 30 min, dehydrated in anhydrous ethanol, air-dried, and mounted with neutral gum. Finally, the slides were examined and photographed under a microscope.

#### Immunohistochemistry staining

After deparaffinization, antigen retrieval was performed. Sections were incubated with 3% hydrogen peroxide at room temperature, rinsed with PBS, and blocked with 2% goat serum. They were then incubated overnight at 4 °C with the primary antibody, followed by incubation with the secondary antibody at 37 °C. Slides were developed with DAB, counterstained with Mayer’s hematoxylin for 2 min, rinsed with PBS to restore the blue coloration, and finally mounted. All slides were examined and photographed under a microscope.

### In vitro experiments

#### Cell culture

Human fetal lung fibroblasts (HFL1) were cultured at 37 °C in a humidified atmosphere with 5% CO_2_. Cells were divided into a control group, a model group treated with 5 ng/mL TGF-β1, and OP-D treatment groups co-treated with 5 ng/mL TGF-β1 and OP-D at 1, 5, or 10 µM to assess the inhibitory effect of OP-D on FTH1 expression.

#### Cell viability assay and cell transfection

HFL1 cells were seeded in 96-well plates at 4500 cells per well and treated for 24 h with OP-D (1, 5, or 10 µM), with or without deferoxamine (DFO). CCK-8 solution was then added, and plates were incubated at 37 °C for 4 h before measuring absorbance at 450 nm.

HFL1 cells were transfected with an FTH1 plasmid (Bio-Tower Biotechnology, Wuhan, China). Cells were plated at 5 × 10^5^ cells per well in 6-well plates. Lipofectamine^™^ 2000 (Invitrogen) (5 µL) was diluted in 100 µL Opti-MEM and incubated briefly. The diluted reagent (200 µL) was added to each well and gently mixed. Plates were incubated at 37 °C with 5% CO₂ for 6 h, after which the transfection mixture was removed and replaced with standard culture medium. Cells were then incubated for an additional 48 h before analysis.

#### Detection of glutathione (GSH) and malondialdehyde (MDA)

Cells were collected and washed three times with PBS. Pellets corresponding to 1 × 10⁶ cells were resuspended in 200 µL PBS and lysed by repeated freeze–thaw cycles. Lysates were centrifuged at 3000 rpm for 10 min, and the supernatant was used to determine protein concentration. MDA and GSH assays were then performed following the manufacturers’ protocols.

#### Detection of reactive oxygen species (ROS)

Approximately 3 × 10^5^ cells were seeded in a 60 mm culture dish. After treatment, the medium was replaced with serum-free medium containing 10 µM DCFH-DA and incubated at 37 °C for 20 min. Cells were then washed twice with PBS to remove excess DCFH-DA and resuspended in PBS for analysis. Fluorescence was measured with excitation at 488 nm and emission at 525 nm, and results were expressed as fluorescence intensity.

#### Detection of apoptosis and ferrous ion concentration by flow cytometry

HFL1 human embryonic lung fibroblasts were thawed and cultured in medium containing 90% F-12 K and 10% FBS. Cells were subcultured at a 1:3 split ratio, and logarithmically growing cells were seeded at 5 × 10^5^ cells per well in 6-well plates and incubated overnight at 37 °C with 5% CO₂. After 24 h, apoptosis was evaluated using Annexin V-FITC/PI staining kits and FerroOrange.

#### TUNEL assay

After deparaffinization, sections were incubated with proteinase K solution and then with TUNEL detection solution at 37 °C. Following PBS washes, nuclei were stained with DAPI. Finally, slides were mounted with sealing medium and examined and photographed under a fluorescence microscope (Olympus, Tokyo, Japan).

#### Transmission electron microscopy (TEM)

Cell specimens were fixed in 3% glutaraldehyde for 3–4 h and then post-fixed in 1% osmium tetroxide at room temperature. After dehydration, ultrathin Sects. (60–80 nm) were cut and double-stained with uranyl acetate and lead citrate. Finally, samples were examined and imaged using a Hitachi-7700 transmission electron microscope (Japan).

#### Immunofluorescence staining

After deparaffinization, antigen retrieval was performed and sections were rinsed with PBS. Sections were blocked with goat serum at room temperature, then incubated overnight at 4 °C with the appropriately diluted primary antibody, followed by three washes with PBST. Next, sections were incubated at 37 °C with a fluorescent secondary antibody. Nuclei were stained with DAPI (Beyotime, Shanghai, China) in the dark, followed by four PBST washes. Finally, slides were mounted with a sealing medium and imaged using a fluorescence microscope (Olympus, Tokyo, Japan).

### Omics analyses and molecular docking

#### Transcriptomic analysis

Total RNA from lung tissues and lung fibroblasts was extracted with TRIzol reagent (Life Technologies, Carlsbad, CA, USA). Poly(A) mRNA was enriched using oligo(dT) magnetic beads, then fragmented at high temperature and reverse-transcribed into double-stranded cDNA. Libraries were prepared by end repair, A-tailing, and adapter ligation, after which target fragments were selected with Hieff NGS^®^ DNA Selection Beads and amplified by PCR. Finally, sequencing was carried out on an Illumina NovaSeq X Plus platform.

#### Proteomic analysis

After grinding the lung tissues, 50 µL of buffer was added and the samples were mixed at 1000 rpm for 10 min. After cooling, trypsin digestion buffer was added and incubated for 2 h, followed by sample purification. Peptides were eluted twice with 100 μL of elution buffer and freeze-dried in a SpeedVac. High-pH reversed-phase separation was then performed, and the data were searched against the UniProt (or provided) database using Spectronaut 18 (Biognosys AG, Switzerland) for DIA analysis with dynamic iRT retention time prediction.

#### Molecular docking

OP-D data were retrieved from PubChem (https://pubchem.ncbi.nlm.nih.gov/), and the crystal structures of Ferritin Heavy Chain 1 (FTH1), Glutathione Peroxidase 4 (GPX4), and Solute Carrier Family 7 Member 11 (SLC7A11) were downloaded from the RCSB PDB database (https://www.rcsb.org/). Protein preparation and optimization, as well as molecular docking, were performed in Schrödinger Maestro. Docking and virtual screening were carried out using the standard precision (SP) protocol.

### Validation experiments

#### Western blot

Total protein was extracted from 10^7^ cells or 30–50 mg of tissue using PMSF-containing protein extraction reagent (Servicebio, Wuhan, China), and concentration was determined by BCA assay (P0010, Beyotime). Proteins were denatured, separated by SDS-PAGE, and transferred to a PVDF membrane. The membrane was incubated with primary antibody overnight at 4 °C, followed by secondary antibody incubation, color development, and imaging. Band intensities were quantified using IPP. Antibody details are listed in Table 2.

**Table 2:** Antibodies used in this study

Antibody	Brand	Cat No	Applications
Collagen I	Sanying Biotechnology	14695-1-AP	1:2000
GPX4	Sanying Biotechnology	67763-1-Ig	1:4000
α-SMA	Abcam	80008-1-RR	1:50,000
TGF-β1	Sanying Biotechnology	21898-1-AP	1:2000
TGF-βR1	Abcam	ab31013	1:1000
SMAD2	abcam	ab33875	1:1000
p-SMAD2	abcam	ab280888	1:1000
SMAD3	abcam	ab40854	1:2000
p-SMAD3	abcam	ab52903	1:2000
SLC7A11	Abcam	26864-1-AP	1:1000
Vimentin	Affinity Biosciences	AF7013	1:1000
FTH1	Boster	BM4487	1:1000
Transferrin	Sanying Biotechnology	17435-1-ap	1:2000
β-actin	Affinity Biosciences	T0022	1:20,000

#### PCR assay

Total RNA was extracted from tissue and cell samples using TRIzol, and RNA purity and concentration were assessed by measuring OD₆₀₀, OD₂₈₀, and the OD₂₆₀/OD₂₈₀ ratio with a spectrophotometer. RNA was then reverse-transcribed into cDNA according to the manufacturer’s instructions, and quantitative real-time PCR was performed using 2 × Q3 SYBR qPCR Master Mix (TOLOBIO). Primer sequences are listed in Table 3.

**Table 3:** Primers used for qPCR in this study

Gene symbol	Primer	Sequence (5′–3′)	Product length
Mus β-actin	Forward	CACGATGGAGGGGCCGGACTCATC	240 bp
	Reverse	TAAAGACCTCTATGCCAACACAGT	
Mus FTH1	Forward	GACCGAGATGATGTGGCTCT	187 bp
	Reverse	GTGCACACTCCATTGCATTC	
Mus GPX4	Forward	AGCCCATTCCTGAACCTTTC	191 bp
	Reverse	GCACACGAAACCCCTGTACT	
Mus SLC7A11	Forward	TTCATCCCGGCACTATTTTC	161 bp
	Reverse	CGTCTGAACCACTTGGGTTT	

### Statistical analysis

All data are presented as mean ± SD. An unpaired Student’s *t*-test was used to compare two groups, and statistical analyses were performed with GraphPad Prism 8.0.2 (GraphPad Software). A *P* value of less than 0.05 was considered statistically significant.

## Results

### Identification of the chemical constituents in MMDD

**Fig. 1:**
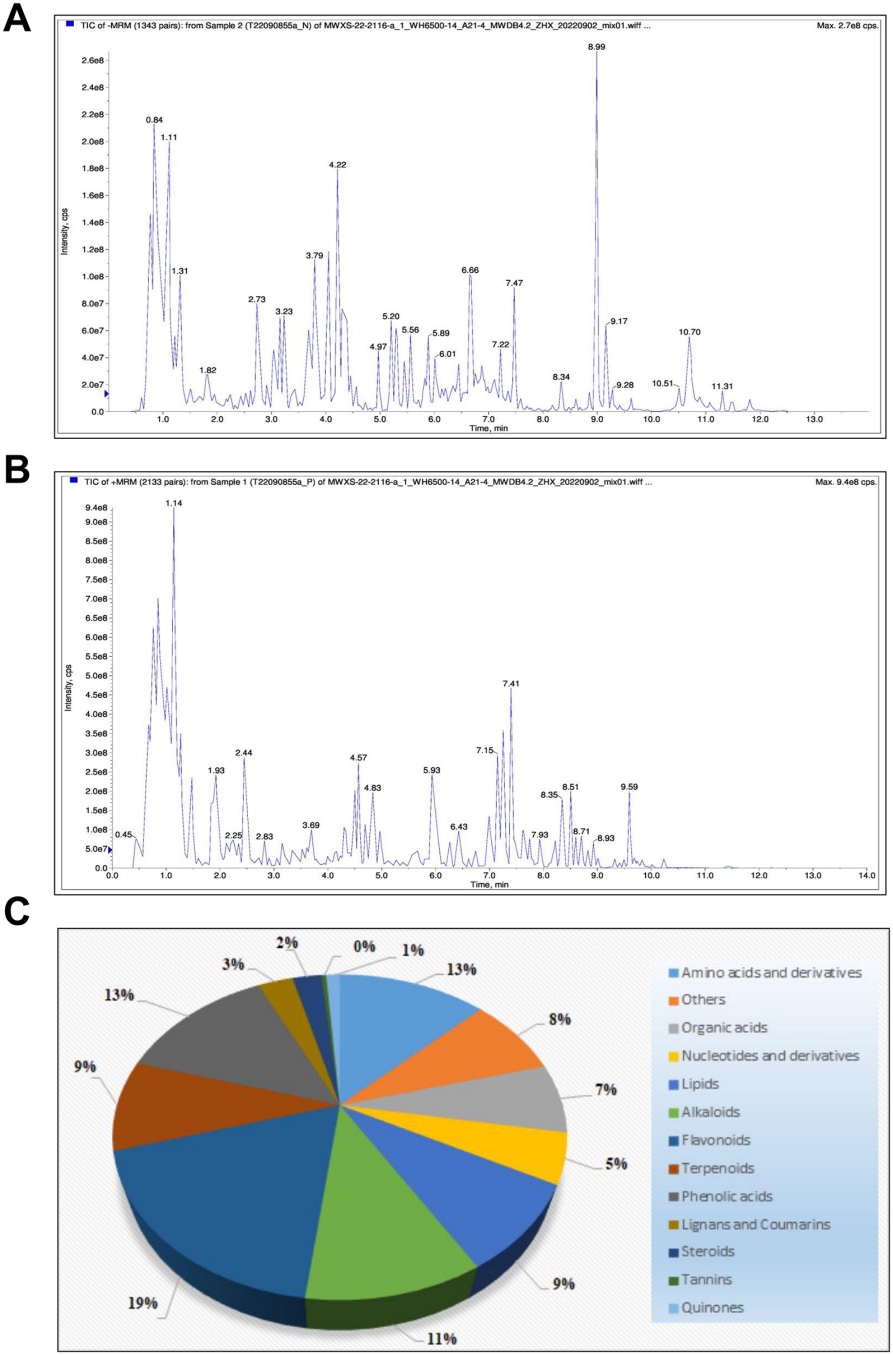
HPLC and UPLC-MS/MS analysis of MMDD. **A** Total ion chromatogram of MMDD in positive mode. **B** Total ion chromatogram of MMDD in negative mode. **C** Count of individual components of MMDD identified by positive and negative UPLC-MS/MS

The total ion chromatograms are shown in Fig. 1A, B. The major compound classes in MMDD include amino acids and derivatives (13%), phenolic acids (13%), alkaloids (11%), terpenoids (9%), organic acids (7%), nucleotides and derivatives (5%), lipids (9%), flavonoids (19%), lignans and coumarins (3%), steroids (2%), and quinones (1%) (Fig. 1C and Table 4).

**Table 4:** Classification and Relative Proportions of Chemical Compounds in MMDD

Chemical class	Percentage (%)
Flavonoids	19
Amino acids and derivatives	13
Phenolic acids	13
Alkaloids	11
Terpenoids	9
Lipids	9
Organic acids	7
Nucleotides and derivatives	5
Lignans and Coumarins	3
Steroids	2
Quinones	1

The data represent the relative proportions of chemical compound categories identified in MMDD using UPLC–MS/MS, as illustrated in Fig. 1C

### MMDD alleviates BLM-induced lung injury and fibrotic lesions

**Fig. 2:**
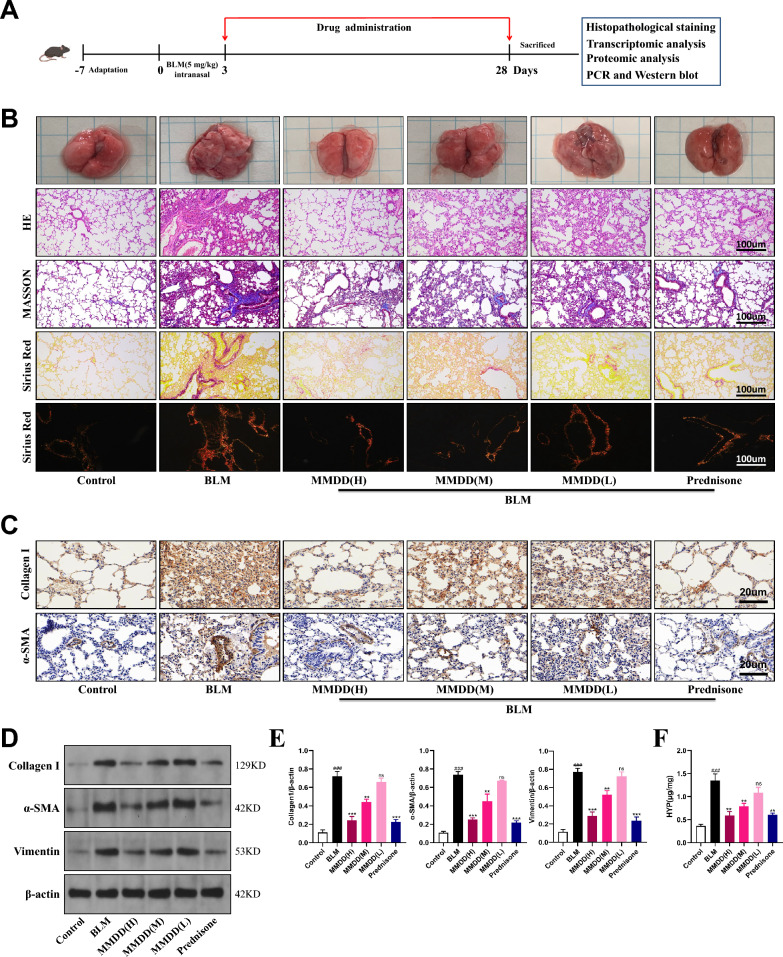
MMDD reduces the severity of BLM-induced pulmonary fibrosis in mice. **A** Experimental outline. Mice were treated with bleomycin via intratracheal instillation at a dose of 5 mg/kg to induce pulmonary fibrosis. Subsequently, MMDD at doses of 17.16 mg/kg, 8.58 mg/kg, or 4.29 mg/kg, along with Prednisone at a dose of 5 mg/kg, was administered to the mice through daily oral gavage until the end of the 28-day experimental period. **B** Representative micrographs of lung tissue sections from all experimental groups, showing H&E, Masson, and Sirius red stains. Scale bar = 100 μm. **C** Immunohistochemistry was performed to assess the protein levels of Collagen I and α-SMA in lung tissue. Scale bar = 20 μm. **D**, **E** Western blotting was performed to quantify the protein levels of Collagen I and α-SMA, with *n* = 3. **F** The hydroxyproline content in lung tissue was quantified (ng/mg). Data expressed as mean ± SD; ###, *p* < 0.001 versus Control group; ***, *p* < 0.001, **, *p* < 0.01, ns, not significant versus BLM group

To assess the therapeutic effects of MMDD on lung injury and fibrotic lesions, we administered varying doses of MMDD to BLM-induced IPF mice (Fig. 2A). Prednisone served as a positive control to compare efficacy, as it has been shown to reduce inflammation and fibrosis in this model [7, 15]. Higher MMDD concentrations produced marked reductions in lung congestion, edema, inflammatory cell infiltration, and collagen accumulation, indicating a dose dependent effect (Fig. 2B). Immunohistochemical staining showed that MMDD significantly inhibited collagen I and α-SMA expression (Fig. 2C). Western blotting confirmed that high-dose MMDD (MMDD-H) significantly lowered protein levels of collagen I, α-SMA, and vimentin (Fig. 2D, E). Furthermore, hydroxyproline assays demonstrated that MMDD-H significantly reduced collagen deposition in lung tissues (Fig. 2F). Together, these findings indicate that MMDD alleviates lung tissue injury and fibrotic lesions in mice.

In addition, we compared the efficacy of MMDD when administered at different time points after BLM induction (Fig. S1a). When initiated on day 7, MMDD treatment produced modest improvements in histopathological features, including attenuation of lung injury and collagen deposition, but these effects were less pronounced than those observed with day 3 administration (Fig. S1b). Immunohistochemical staining for collagen I and α-SMA, as well as hydroxyproline measurements, showed consistent trends (Fig. S1c, d). Based on these findings, subsequent analyses in this study were performed using tissue samples from animals treated at the day-3 time point.

### MMDD can intervene in the progression of fibrosis by inhibiting TGF-β signal transduction

**Fig. 3:**
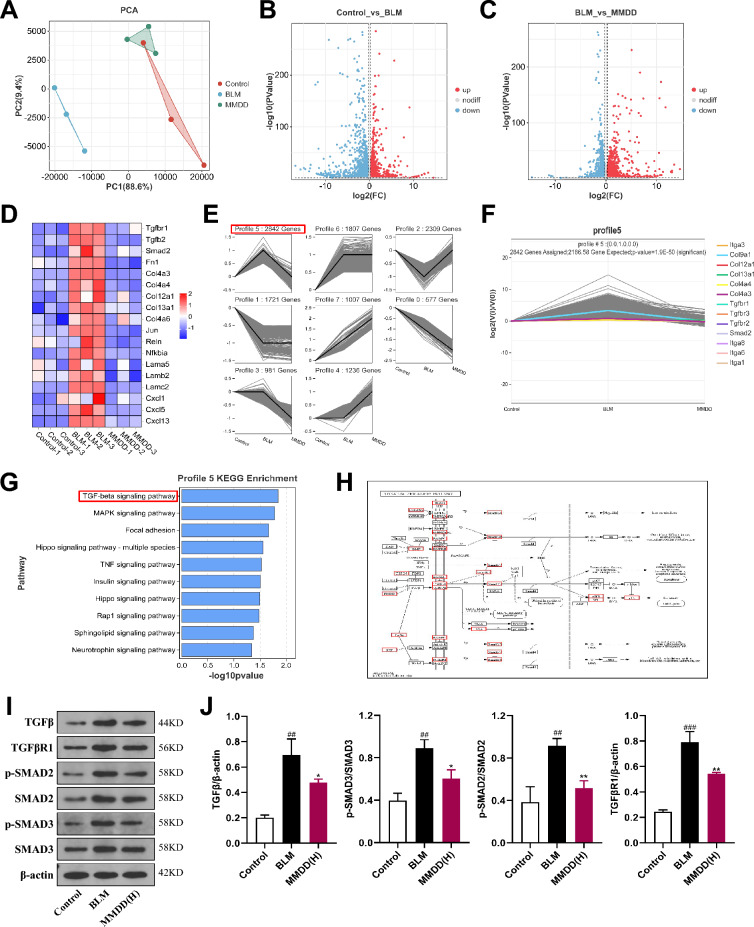
Changes in the transcriptome of pulmonary fibrosis upon MMDD intervention. **A** Principal component analysis of RNA-seq data from the Control, BLM, and MMDD groups. **B** Volcano plot of differentially expressed genes (DEGs) between Control and BLM. **C** Volcano plot of DEGs between BLM and MMDD-H. **D** Heatmap representing the expression of ferroptosis-associated genes in transcriptomic data. **E**, **F** Transcriptomic differential gene trend analysis was divided into eight modules, with the fifth module enriched with 2842 genes. **G** KEGG enrichment analysis for the genes of Module 5. **H** KEGG pathway map of TGF-β signaling, where red boxes highlight a reduction in the BLM group and an elevation following MMDD treatment. **I**, **J** Western blot analysis and quantitative assessment of protein expression for TGF-β, TGFβR1, p-Smad2, Smad2, p-Smad3 and Smad3, with *n* = 3. Data expressed as mean ± SD; ###, *p* < 0.001, ##, *p* < 0.01 versus Control group; **, *p* < 0.01, *, *p* < 0.05 versus BLM group

To further investigate the genetic changes induced by MMDD in pulmonary fibrosis, we performed transcriptomic analysis on lung tissues from control, bleomycin (BLM)–treated, and high-dose MMDD (MMDD-H) mice. Principal component analysis revealed three distinct clusters corresponding to the three groups, with a clear separation between the BLM and MMDD-H groups (Fig. 3A). Compared with control, the BLM group exhibited 3131 upregulated and 2557 downregulated genes; after MMDD treatment, 1994 genes were upregulated and 1921 were downregulated (*p* < 0.05, |log₂FC|> 1.2; Fig. 3B, C). Heatmap analysis showed that fibrosis- and inflammation-related genes—such as Tgfbr1, Smad3, Col4a3, Nfkbia, and Cxcl1—were markedly reduced following MMDD intervention (Fig. 3D). Trend analysis identified Module 5, comprising 2842 genes that increased after BLM treatment and decreased after MMDD administration (Fig. 3E, F). KEGG pathway analysis revealed significant enrichment of this module in the TGF-β signaling pathway (Fig. 3G), and many TGF-β pathway genes upregulated by BLM were significantly reversed by MMDD (Fig. 3H). Western blot analysis further confirmed that MMDD-H significantly reduced protein levels of TGF-β, TGF-βR1, SMAD2, p-SMAD2, SMAD3, and p-SMAD3 (Fig. 3I, J). Overall, these findings demonstrate that MMDD effectively inhibits activation of the TGF-β signaling pathway in a mouse model of pulmonary fibrosis.

### Proteomics and integrated transcriptome-proteome correlation analysis

**Fig. 4:**
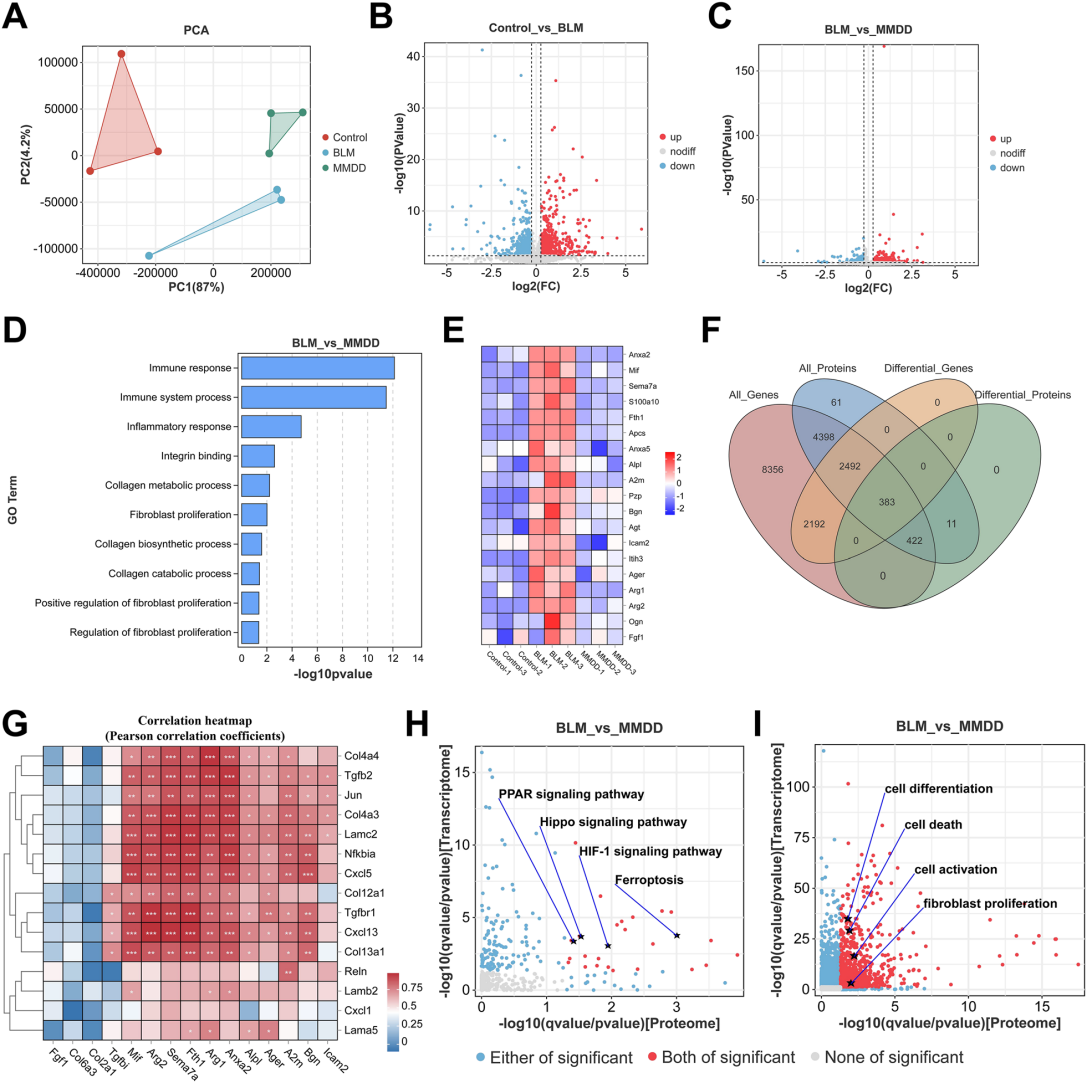
Proteomics analysis and integrated transcriptome-proteome analysis. **A** PCA analysis of proteomic data for Control, BLM, and MMDD groups. **B** DEPs volcano map of Control versus BLM. **C** DEPs volcano map of BLM versus MMDD-H. **D** GO enrichment analysis of differential proteins in the BLM and MMDD-H groups. **E** Heat map of ferroptosis and inflammation-related protein expression in proteomics. **F** Venn diagram illustrating the gene intersections between transcriptomics and proteomics. **G** Pearson correlation heatmap showing transcriptome–proteome expression correlations for MMDD-regulated genes and fibrosis-related markers. Red indicates positive and blue negative correlations. Asterisks denote significance levels (*p < 0.05, **p < 0.01, ***p < 0.001). **H**, **I** Analysis of the correlation between GO terms and KEGG pathways in transcriptomic and proteomic data

The TGF-β signaling pathway is closely associated with fibroblast proliferation and activation. To elucidate MMDD’s mechanism, we conducted proteomic sequencing and integrated transcriptome–proteome correlation analysis. Proteomic PCA showed distinct clustering of the control, BLM, and MMDD groups (Fig. 4A). Compared with control, the BLM group exhibited 681 upregulated and 768 downregulated proteins, whereas MMDD treatment resulted in 358 upregulated and 458 downregulated proteins (*p* < 0.05, |log₂FC|> 1.2; Fig. 4B, C). Functional enrichment analysis revealed significant enrichment of immune response, fibroblast differentiation, and collagen synthesis (Fig. 4D). Heatmap analysis indicated that pro-fibrotic and pro-inflammatory proteins, including ICAM2, FTH1, MIF, and ITIH3, were markedly downregulated following MMDD intervention (Fig. 4E).

Integrated transcriptome–proteome analysis identified 383 differentially expressed genes and proteins (Fig. 4F). To explore coordinated regulation between MMDD targets and fibrosis-related genes, Pearson correlation analysis was performed (Fig. 4G). The heatmap showed that OP-D–modulated genes—including Fth1, Mif, Sema7a, and Arg1—were strongly correlated with key fibrotic markers such as Tgfb2, Tgfbr1, Col4a3, and Lamc2, suggesting co-regulation within fibrotic signaling networks. Pathway and GO enrichment analyses of these differentials revealed significant enrichment in ferroptosis-related pathways, including ferroptosis itself, PPAR, Hippo, and HIF-1 signaling (Fig. 4H). In addition, cellular processes such as fibroblast proliferation, differentiation, death, and activation were markedly regulated by MMDD (Fig. 4I). In summary, MMDD ameliorates pulmonary fibrosis by modulating fibroblast proliferation and ferroptosis through inhibition of the TGF-β signaling pathway.

### Ophiopogonin-D is capable of restraining the expansion and activation of lung fibroblasts

**Fig. 5:**
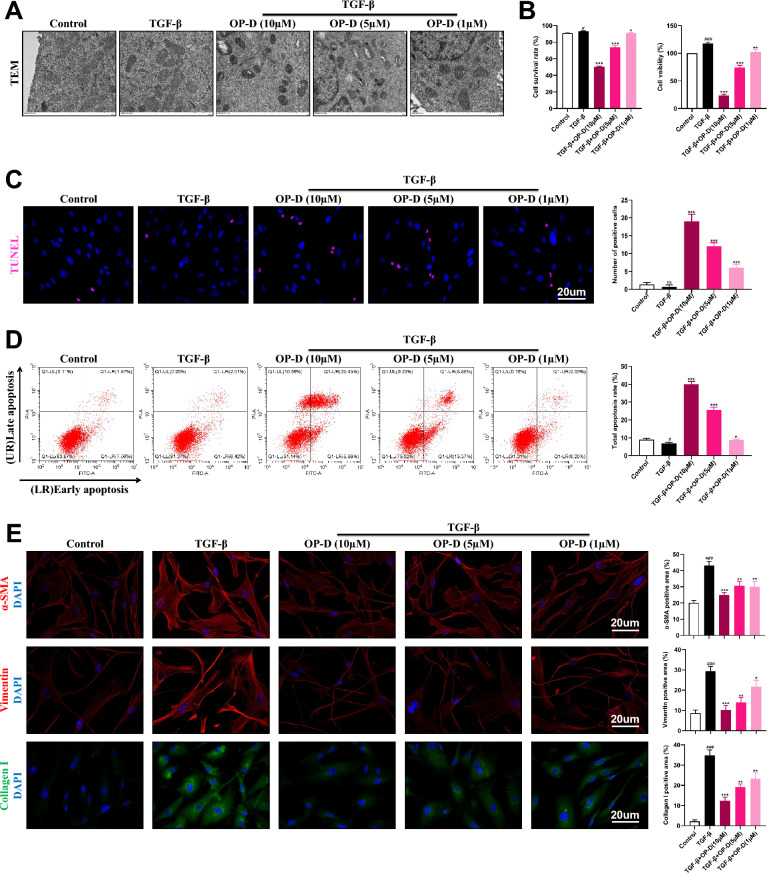
Ophiopogonin-D (OP-D) blocks fibroblast proliferation and activation, simultaneously increasing apoptosis. **A** Transmission electron microscopy images showing changes in mitochondria in TGF-β-treated HFL1 cells with or without OP-D treatment, Scale bar = 1 μm. **B** Cell proliferation rate was assessed using CCK8. **C**, **D** TUNEL assays were conducted to evaluate apoptosis. **E** Immunofluorescence staining was used to detect the colocalization of α-SMA (red), Vimentin (red), and Collagen I (green) in DAPI (blue)-positive lung fibroblasts, with quantification of immunolabeled areas across treatment groups, Scale bar = 20 μm. Data expressed as mean ± SD; ###, *p* < 0.001, #, *p* < 0.05, ns, not significant versus Control group; ***, *p* < 0.001, **, *p* < 0.01, *, *p* < 0.05 versus TGF-β group

OP-D, a principal active component of MMDD, exhibits significant anti-fibrotic activity in TGF-β–induced fibroblasts. We hypothesized that OP-D is the main bioactive constituent of MMDD and exerts its effects by modulating lung fibroblast behavior. To test this, TGF-β–induced HFL1 cells were treated with increasing concentrations of OP-D to elucidate its impact on fibroblast function. Transmission electron microscopy revealed that OP-D reduced mitochondrial volume and increased membrane density (Fig. 5A). CCK-8 assays showed a dose-dependent decrease in cell viability and survival rates (Fig. 5B). TUNEL staining demonstrated a significant rise in positive cells following OP-D treatment (Fig. 5C), and flow cytometry confirmed dose-dependent induction of apoptosis (Fig. 5D). Immunofluorescence analysis further revealed dose-dependent reductions in α-SMA and vimentin expression, along with decreased Collagen I levels and collagen deposition (Fig. 5E). Together, these results indicate that OP-D induces fibroblast death while inhibiting cell activation and collagen accumulation, underscoring its pivotal role as the main active component of MMDD in regulating lung fibroblast behavior and attenuating fibrosis progression.

### Ophiopogonin-D can promote ferroptosis in lung fibroblasts

**Fig. 6:**
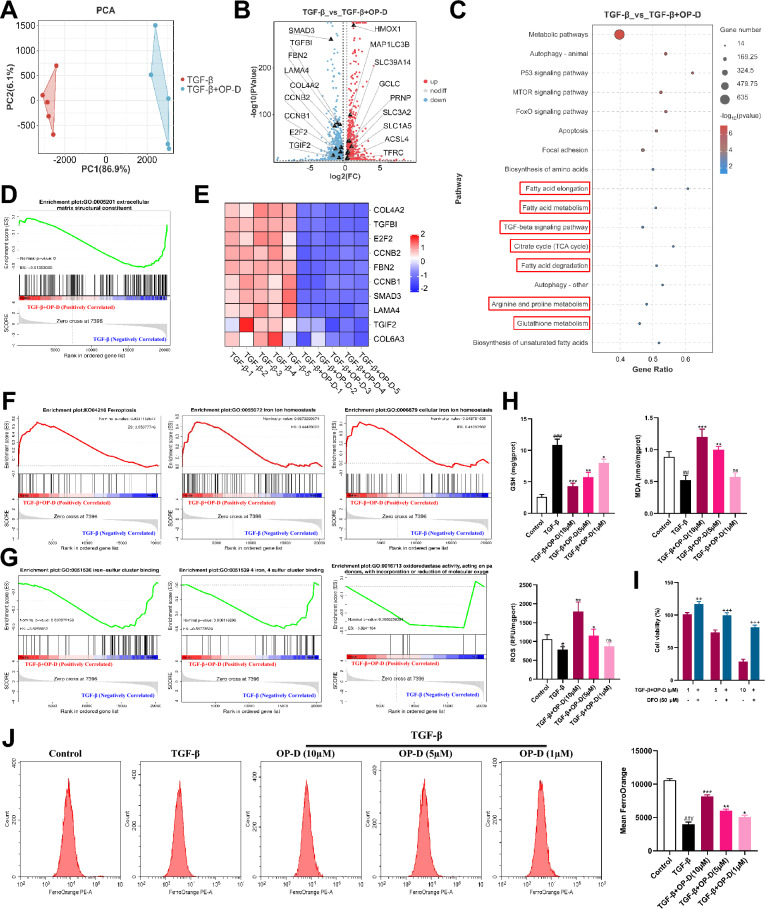
OP-D promotes ferroptosis within lung fibroblasts. **A** The PCA analysis of RNA-seq data was performed for the TGF-β and TGF-β + OP-D-H groups. **B** Differentially expressed genes were statistically analyzed, with red representing upregulation and blue representing downregulation. **C** KEGG pathway enrichment analysis for the differentially expressed genes. **D** GSEA analysis of extracellular matrix structural components in cells from different treatment groups. **E** Heatmap of fibrosis-related gene expression before and after OP-D intervention. **F** GSEA analysis of ferroptosis and iron homeostasis-related gene modules in cells from different treatment groups. **G** GSEA analysis of iron-sulfur cluster binding and oxidoreductase activity-related gene modules in cells from different treatment groups. **H** Levels of ROS, GSH, and MDA in lung fibroblasts after OP-D intervention. **I** Cell viability of lung fibroblasts after treatment with OP-D alone or in combination with deferoxamine (DFO) for 24 h. **J** Detection of intracellular ferrous ion content by flow cytometry. Data expressed as mean ± SD; ###, *p* < 0.001, ##, *p* < 0.01, #, *p* < 0.05 versus Control group; ***, *p* < 0.001, **, *p* < 0.01, *, *p* < 0.05, ns, not significant versus TGF-β group; + + +, *p* < 0.001, + +, *p* < 0.01 versus TGF-β + OP-D group

To further elucidate the type of cell death induced by OP-D in lung fibroblasts, we performed transcriptomic sequencing on HFL1 cells treated with TGF-β alone or with TGF-β plus 10 µM OP-D. Principal component analysis revealed clear separation between the two groups (Fig. 6A). Volcano plot analysis showed that pro-fibrotic and anti-ferroptosis genes (e.g., TGFBI, SMAD3, COL4A1, FTH1, and GPX4) were significantly downregulated by OP-D, whereas pro-ferroptosis genes (e.g., TFRC, ACSL4, and GCLC) were markedly upregulated (*p* < 0.05, |log₂FC|> 1.2; Fig. 6B). Pathway enrichment revealed significant activation of the TGF-β and ferroptosis pathways after OP-D treatment (Fig. 6C). Building on our previous finding that OP-D suppresses TGF-β signaling to modulate fibroblast proliferation and differentiation [7], we next examined specific modes of cell death. Gene set enrichment analysis showed a pronounced decrease in extracellular matrix structural component genes following OP-D intervention (Fig. 6D, E) and enrichment of ferroptosis and iron ion homeostasis pathways (Fig. 6F). OP-D also significantly inhibited gene sets related to iron–sulfur cluster binding and associated oxidoreductase activities, suggesting it elevates free iron levels and disrupts redox balance, thereby sensitizing cells to ferroptosis (Fig. 6G). Consistently, biochemical assays demonstrated that high concentrations of OP-D reduced GSH levels while increasing MDA and ROS (Fig. 6H). Inhibition experiments using DFO significantly rescued OP-D–induced cell death (Fig. 6I), and flow cytometry confirmed a dose-dependent increase in intracellular Fe^2^⁺ levels with OP-D treatment (Fig. 6J). Together, these data indicate that OP-D induces ferroptosis in lung fibroblasts by disrupting iron homeostasis and impairing iron–sulfur cluster binding, thereby enhancing cellular sensitivity to ferroptosis.

### Ophiopogonin-D suppresses the anti-ferroptotic regulatory signals mediated by FTH1 in lung fibroblasts

**Fig. 7:**
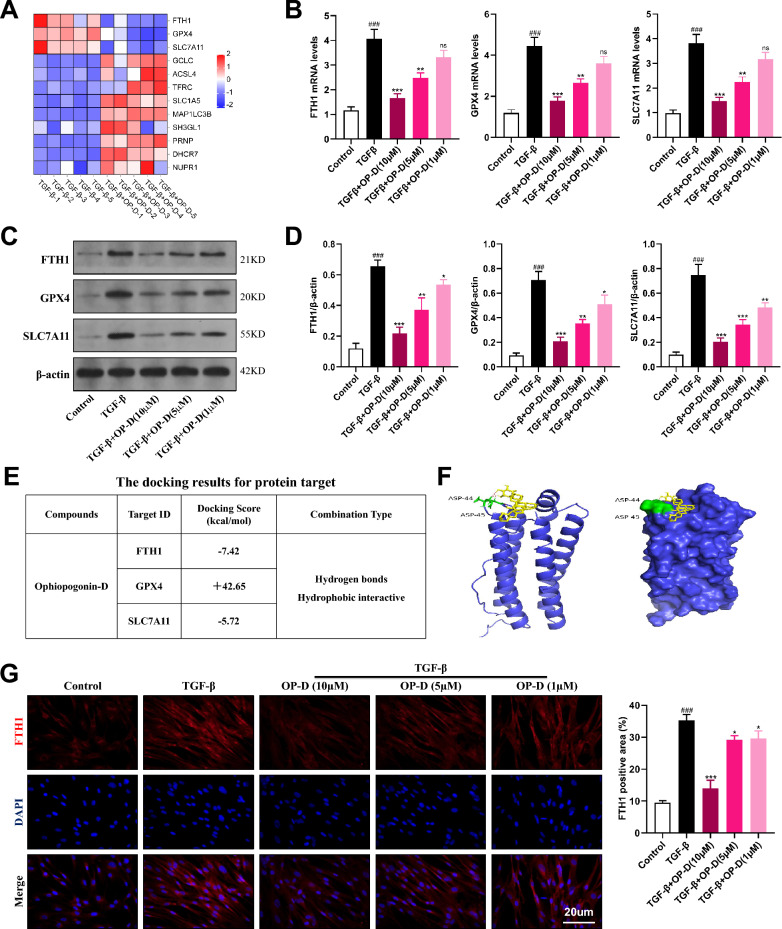
FTH1 serves as a crucial target for OP-D in promoting ferroptosis in lung fibroblasts. **A** Heatmap of ferroptosis-related protein expression before and after OP-D treatment. **B** The mRNA expression of FTH1, GPX4, and SLC7A11 in different cell groups, n = 3. **C**, **D** Western blotting and quantification of protein expression for FTH1, GPX4, and SLC7A11 in diverse cell groups. **E** Binding Efficiency of the OP-D to FTH1, GPX4 and SLC7A11 Molecules. **F** The interaction pattern between the FTH1 protein and OP-D. **G** Immunofluorescence colocalization of FTH1 in human lung fibroblasts treated with varying concentrations of OP-D, with quantification of immunolabeled areas across treatment groups, Scale bar = 20 μm. Data expressed as mean ± SD; ###, *p* < 0.001 versus Control group; ***, *p* < 0.001, **, *p* < 0.01, *, *p* < 0.05, ns, not significant versus TGF-β group

To clarify how OP-D induces ferroptosis in lung fibroblasts, we performed additional transcriptomic analyses. Heatmap results showed a pronounced reduction in FTH1, GPX4, and SLC7A11 expression following OP-D treatment, indicating that OP-D substantially disrupts the negative regulatory mechanisms of ferroptosis in these cells (Fig. 7A). We then validated these findings by western blot and RT-qPCR, which confirmed that OP-D decreased both mRNA and protein levels of FTH1, GPX4, and SLC7A11 in a dose-dependent manner (Fig. 7B–D). To identify the key anti-ferroptotic target, we conducted molecular docking of OP-D with FTH1, GPX4, and SLC7A11. The binding energy between OP-D and FTH1 was − 7.42 kcal/mol, substantially lower than those for GPX4 and SLC7A11, suggesting a more stable interaction with FTH1 (Fig. 7E). The molecular interactions are illustrated in Fig. 7F. Finally, immunofluorescence analysis confirmed that OP-D reduces FTH1 expression in lung fibroblasts (Fig. 7G). Together, these data suggest that OP-D promotes ferroptosis primarily by suppressing FTH1-mediated negative regulation of this cell death pathway.

### FTH1 is the key determinant in ophiopogonin-D-induced ferroptosis in lung fibroblasts

**Fig. 8:**
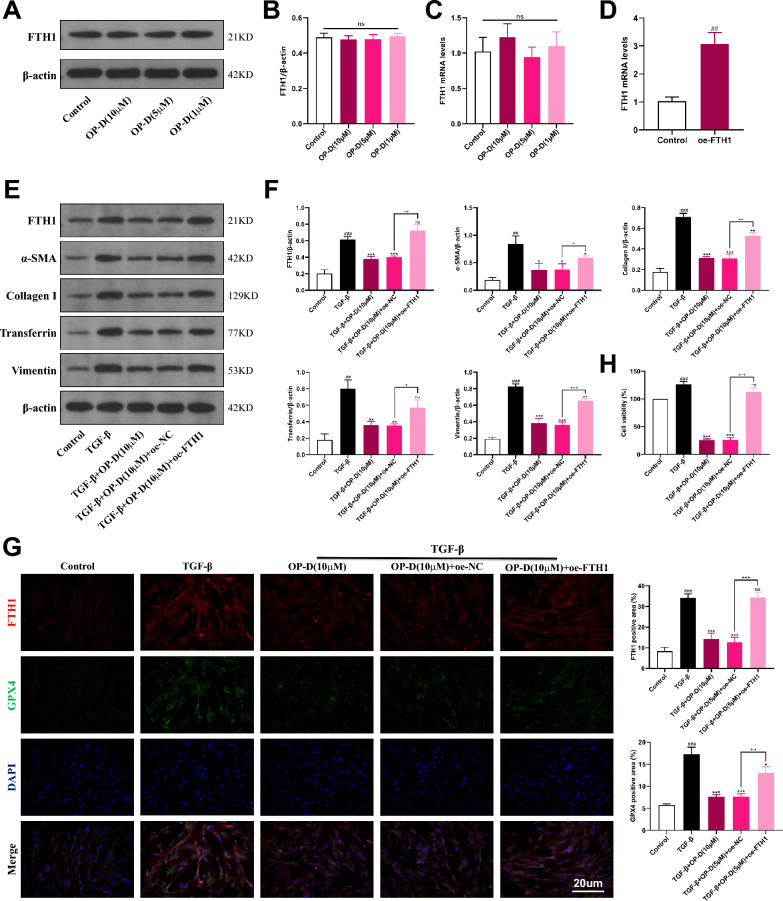
Overexpression of FTH1 can eliminate the effect of OP-D in promoting ferroptosis in lung fibroblasts. **A**, **B** Western blotting and quantitative evaluation of how varying concentrations of OP-D influence FTH1 protein expression in HFL1 cells without TGF-β stimulation. **C** The mRNA expression of FTH1 in different cell groups. **D** The qPCR detection of the mRNA expression after overexpression of FTH1. **E**, **F** Western blotting and the quantitative evaluation of the protein expressions of FTH1, α-SMA, Collagen I, Transferrin, and Vimentin after the overexpression of FTH1. **G** Cell proliferation rates were assessed using the CCK8 assay. **H** Immunofluorescence analysis showed colocalization of FTH1 and GPX4, with quantification of their immunolabeled areas across different treatment groups, Scale bar = 20 μm. Data expressed as mean ± SD; ###, *p* < 0.001, ##, *p* < 0.01, ns, not significant versus Control group; ***, *p* < 0.001, **, *p* < 0.01, *, *p* < 0.05, ns, not significant versus TGF-β group. + + +, *p* < 0.001, + +, *p* < 0.01, +, *p* < 0.05 versus TGF-β + OP-D (10 μM) + oe-NC group

To assess whether FTH1 plays an important role in OP-D-induced ferroptosis, we first treated HFL1 cells with different concentrations of OP-D in the absence of TGF-β intervention; the results showed that OP-D had no significant effect on FTH1 expression in non-activated HFL1 cells (Fig. 8A-C). Subsequently, we transfected HFL1 cells with an siRNA plasmid (Fig. 8D), and the results revealed that FTH1 overexpression markedly elevated the expression of Transferrin, α-SMA, Vimentin, and Collagen I (COL I) (Fig. 8E, F). Additionally, immunofluorescence analysis confirmed that FTH1 overexpression also led to a significant increase in GPX4 expression (Fig. 8G). CCK8 assay results showed that FTH1 overexpression abolished the inhibitory effect of OP-D on cell viability (Fig. 8H). These findings indicate that FTH1 is a key regulatory factor in OP-D-induced ferroptosis in lung fibroblasts.

## Discussion

The incidence and mortality of IPF have risen continuously in recent years. Traditional Chinese medicine has been shown to offer additional treatment options for IPF, with advantages such as fewer side effects and lower costs [16]. Maimendong Decoction (MMDD) is widely used in clinical practice for respiratory diseases including cough, lung cancer, and pulmonary fibrosis, yet its clinical application is limited by unclear active components and mechanisms of action [14]. Therefore, we employed a bleomycin-induced IPF mouse model to investigate MMDD’s mechanisms and potential targets, aiming to provide a basis for further research and clinical application.

In this study, we first examined the bioactive components of MMDD and identified multiple categories, including terpenoids, flavonoids, phenolic acids, amino acids and derivatives, and lipids. These classes of compounds are well known for their diverse pharmacological activities, encompassing antioxidant, anti-inflammatory, and anti-fibrotic effects. In the context of IPF, such bioactivities can collectively contribute to the suppression of inflammatory responses, inhibition of fibroblast activation, and reduction of excessive extracellular matrix (ECM) deposition. For instance, flavonoids and phenolic acids are frequently reported to modulate oxidative stress and profibrotic signaling pathways, while terpenoids often exhibit potent immunomodulatory and tissue-protective properties [17–19]. The coexistence of these compound classes in MMDD highlights its potential as a multi-target, multi-pathway therapeutic agent, which may act synergistically to ameliorate fibrosis progression and provides a solid basis for subsequent mechanistic investigations.

Fibroblasts are the main source of extracellular matrix (ECM) and play a crucial role in pulmonary fibrosis progression. Upon activation, they produce abundant ECM, accelerating fibrotic progression [4, 20]. Our findings show that MMDD suppressed α-SMA expression, a marker of fibroblast activation, in IPF mice and concurrently reduced Collagen I, vimentin, and hydroxyproline levels, indicating that MMDD’s therapeutic effect in IPF is closely linked to modulation of fibroblast activity. In addition, we compared the therapeutic effects of MMDD administration at different time points. In the BLM-induced pulmonary fibrosis model, day 3 post-induction corresponded to the peak of the inflammatory response, which substantially overlapped with the initiation of fibrotic progression, representing a critical window for early intervention in fibrosis development [21]. Our results similarly demonstrated that initiating MMDD treatment on day 3 achieved a more pronounced reversal of fibrotic lesions compared to initiation on day 7. Therefore, all subsequent mechanistic analyses in this study were conducted using lung tissues from the day-3 treatment group.

TGF-β is a key signaling molecule in fibrotic processes. As a member of the peptide family, it regulates multiple cellular functions—proliferation, differentiation, and apoptosis—and participates in embryonic lung development, organ homeostasis, and tissue repair [22, 23]. Evidence indicates that activation of the TGF-β pathway induces fibroblast activation, proliferation, and epithelial-to-mesenchymal transition (EMT), expanding the fibroblast pool and contributing to the initiation and progression of various fibrotic diseases. Moreover, activated fibroblasts can increase TGF-β secretion, forming a harmful feedback loop [24, 25]. Recent studies also show that TGF-β signaling affects intracellular iron metabolism and ROS production, thereby influencing ferroptosis [26, 27]. Our macroscopic and histological analyses demonstrate that MMDD significantly alleviates BLM-induced lung tissue damage and collagen accumulation. In addition, our multi-omics and bioinformatics analyses suggest that MMDD’s anti-fibrotic effects are partly due to its ability to modulate fibroblast proliferation and ferroptosis by suppressing the TGF-β signaling pathway.

To investigate MMDD’s mechanism in treating IPF, we conducted in vitro experiments to assess the effects of its primary active ingredient, OP-D, on lung fibroblasts. The results showed that OP-D induced apoptosis and cell death in a dose-dependent manner, thereby exerting anti-fibrotic effects. Ferroptosis—a regulated form of cell death controlled by multiple genes and signaling pathways—has emerged as a promising therapeutic target for tumors and fibrotic diseases. Given that natural compounds are a key resource for anti-fibrotic drug development, those capable of inducing ferroptosis in fibroblasts are attracting increasing interest [28]. An imbalance in iron homeostasis is a critical trigger for ferroptosis: when cellular free iron is low, iron regulatory proteins bind iron-responsive elements (IREs), raising free iron levels and maintaining homeostasis; conversely, excess free iron causes these proteins to associate with iron–sulfur clusters, reducing IRE binding, lowering free iron levels, and preventing ferroptosis [29]. In our study, OP-D modulated lipid metabolism and the ferroptosis pathway and reduced protein–iron–sulfur cluster binding, thereby disrupting iron homeostasis and sensitizing lung fibroblasts to ferroptosis. Moreover, oxidative stress imbalance—a hallmark of ferroptosis tightly regulated by oxidoreductases—was notably affected by OP-D treatment, altering the expression of related gene sets [30]. OP-D also significantly decreased GSH—a key ferroptosis marker—while increasing MDA, ROS, and intracellular Fe^2^⁺. Co-treatment with DFO reversed these changes, further supporting that ferroptosis is the predominant mode of OP-D–induced cell death in pulmonary fibroblasts.

Research indicates that inhibiting anti-ferroptotic regulators in fibroblasts and tumor cells can promote ferroptosis, thereby preventing and treating fibrosis and cancer [31, 32]. Key negative regulators of ferroptosis include FTH1, GPX4, and SLC7A11. FTH1, the heavy-chain subunit of ferritin, is critical for maintaining intracellular iron homeostasis. For example, baicalin induces ferroptosis in tumor cells by inhibiting FTH1, and FTH1 overexpression attenuates baicalin’s anticancer effects in bladder cancer [33]. GPX4 is a GSH-dependent selenoenzyme; its inhibition leads to lipid hydroperoxide accumulation and ferroptosis. In systemic sclerosis, GPX4 inhibition significantly reduces fibroblast viability and increases their sensitivity to ferroptosis [34]. SLC7A11 imports extracellular cystine for GSH synthesis, and its loss lowers GSH levels; in liver fibrosis, SLC7A11 inhibition increases ferroptosis in hepatic stellate cells [31]. In our study, OP-D dose-dependently downregulated FTH1, GPX4, and SLC7A11 to induce ferroptosis in lung fibroblasts. Molecular docking identified FTH1 as a primary OP-D target, implicating it in iron homeostasis regulation, and functional validation confirmed this finding. Notably, OP-D did not affect FTH1 expression in the absence of TGF-β, indicating that its action is context-dependent and specific to fibrotic activation. This selectivity may underlie its therapeutic specificity and safety. Furthermore, FTH1 overexpression abolished OP-D’s anti-fibrotic and pro-ferroptotic effects, confirming its functional importance.

In recent years, increasing attention has been directed toward the development of novel anti-fibrotic agents targeting fibroblasts. In this study, we demonstrated for the first time that MMDD ameliorates fibrotic symptoms in mice with IPF by modulating fibroblast proliferation and ferroptosis through inhibition of the TGF-β signaling pathway. We further confirmed the pivotal role of OP-D, an active component of MMDD, which targets FTH1 to suppress the expression of anti-ferroptotic regulatory genes, thereby promoting ferroptosis in lung fibroblasts and alleviating IPF. Although OP-D exhibited a critical role in our study, MMDD is a multi-component formulation, and other bioactive constituents may also contribute to its anti-fibrotic effects, potentially acting synergistically with OP-D. Flavonoids, for example, have been reported to exert multiple pharmacological actions in pulmonary fibrosis, including antioxidant and anti-inflammatory activities, inhibition of fibroblast activation, and reduction of collagen deposition [17]. Rosmarinic acid, a representative phenolic acid, has been shown to interfere with fibroblast-to-myofibroblast transition and energy metabolism through multiple pathways, thereby delaying fibrosis progression [35]. Eucalyptol, a monoterpenoid, has been reported to suppress inflammation-related signaling and attenuate pulmonary fibrosis [36]. These constituents may complement OP-D in modulating fibroblast activity and extracellular matrix remodeling. Further comprehensive and rigorously designed studies are warranted to elucidate such multi-component synergistic mechanisms and fully characterize the overall pharmacological profile of MMDD.

## Conclusion

**Fig. 9:**
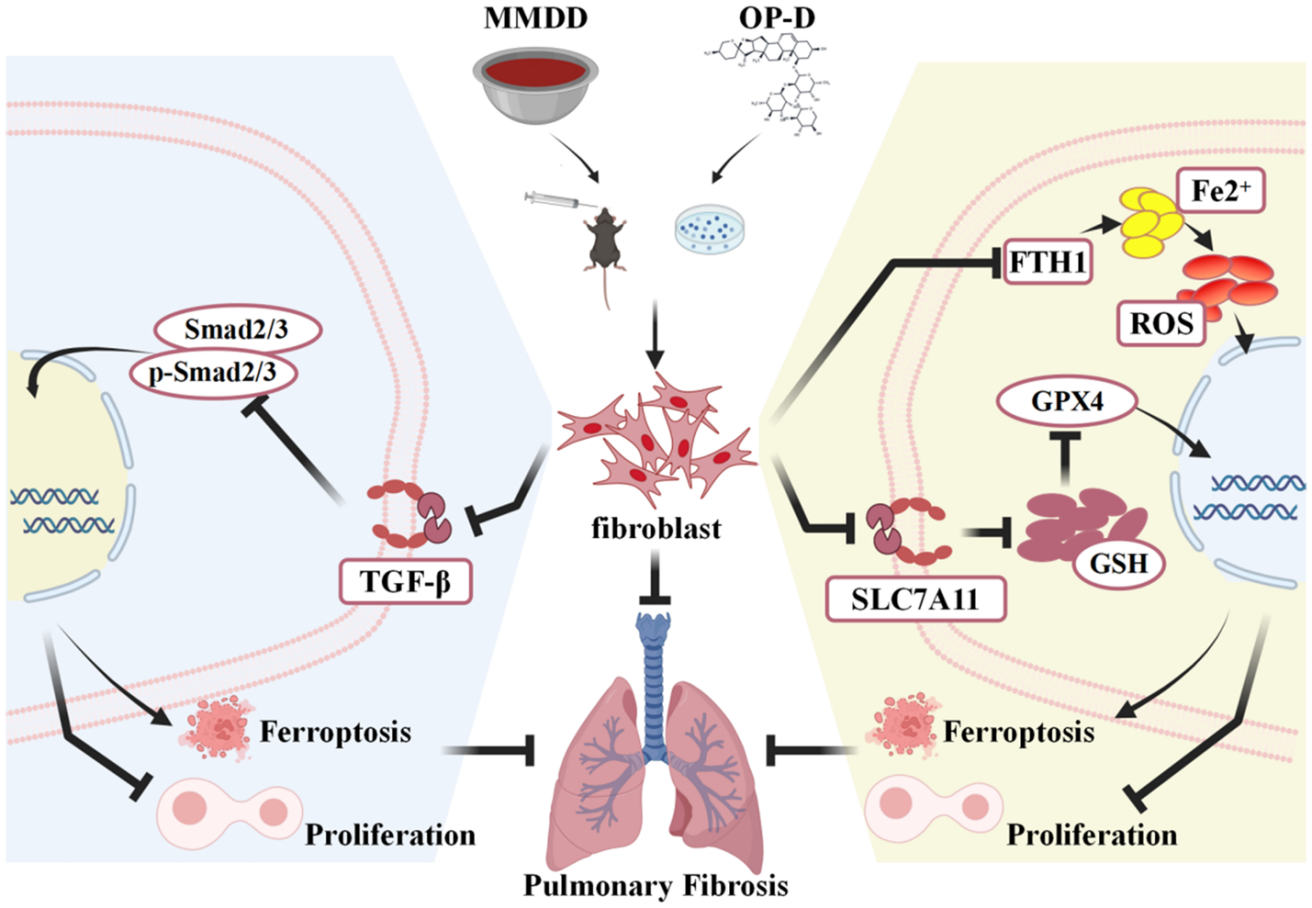
MMDD and its principal active constituent, OP-D, alleviate pulmonary fibrosis through targeting lung fibroblasts. Both MMDD and OP-D exhibit anti-fibrotic properties by inhibiting the TGF-β signaling pathway, reducing fibroblast proliferation, and inducing ferroptosis, while suppression of FTH1 expression is recognized as a critical target

In summary, our results demonstrate that MMDD and its major active component, OP-D, have potential for treating pulmonary fibrosis (Fig. 9). MMDD exerts its anti-fibrotic effects by inhibiting the TGF-β signaling pathway, reducing fibroblast proliferation, and inducing ferroptosis. Importantly, in vitro experiments using OP-D further confirmed the specific mechanism by which MMDD treats pulmonary fibrosis, namely through suppression of anti-ferroptotic regulatory gene expression in lung fibroblasts. In the future, we will continue to explore the mechanisms of other key components in MMDD on pulmonary fibrosis, providing new evidence for the use of traditional Chinese medicine in preventing and treating IPF.

## Supplementary Information

Additional file 1. Data expressed as mean ± SD; ###, p < 0.001 vs. Control group; *, p < 0.05 vs. BLM group.)

## Data Availability

The data associated with this study can be obtained from the corresponding author upon reasonable request.

## Abbreviations

BLM: Bleomycin
ECM: Extra cellular matrix
EMT: Epithelial-mesenchymal transition
FTH1: Ferritin Heavy Chain 1
GPX4: Glutathione Peroxidase 4
GSH: Glutathione
HYP: Hydroxyproline
IPF: Idiopathic pulmonary fibrosis
MDA: Malondialdehyde
MMDD: Maimendong Decoction
OP-D: Ophiopogonin D
ROS: Reactive Oxygen Species
SLC7A11: Solute Carrier Family 7 Member 11
TGF-β: Transforming growth factor beta
TEM: Transmission Electron Microscope
TFRC: Transferrin Receptor
VIM: Vimentin
α-SMA: α-Smooth muscle actin
